# Falsely Elevated Estradiol Levels Due to Heterophile Antibody Interference: A Case Report and Literature Review

**DOI:** 10.2174/0118715303352682250219044719

**Published:** 2025-03-20

**Authors:** Ying Guo, Bin Wei, Wei Dai, Ge Zhang

**Affiliations:** 1 Department of Laboratory Medicine, West China Second Hospital, Sichuan University, Key Laboratory of Birth Defects and Related Diseases of Women and Children (Sichuan University), Ministry of Education, Chengdu, Sichuan Province 610041, China;; 2 Department of Laboratory Medicine/Clinical Research Center of Laboratory Medicine, West China Hospital, Sichuan University, Chengdu, Sichuan Province 610041, China

**Keywords:** Estradiol, heterophile antibody, immunoassay, interference, polyethylene glycol precipitation, dilution test

## Abstract

**Background:**

Immunoassay interference remains an important issue in clinical laboratory medicine, and false increases in estradiol levels due to detection interference are rare, but they cannot be ignored.

**Case Presentation:**

We herein report the case of a 41-year-old woman who underwent a series of extensive and superfluous medical examinations because of erroneous laboratory findings according to the Siemens Centaur serum estradiol assay due to heterophile antibody interference. Her estradiol levels were measured several times, which measured between 7534 and 9772 pg/mL. Heterophile antibody interference was identified through method comparisons, polyethylene glycol precipitation, a gradient dilution test, and the use of a heterophile-blocking reagent.

**Conclusion:**

The rapid identification of interference in estradiol testing poses a challenge for laboratory staff; however, it is vital for preventing unnecessary medical treatment and reducing healthcare costs. Analytical interference should be suspected when clinical manifestations do not align with laboratory results. Consequently, clinicians and laboratory personnel need to develop an anti-interference workflow to mitigate such occurrences in the future.

## 1 INTRODUCTION

We present the complex case of a 41-year-old woman who underwent a series of extensive and unnecessary medical examinations due to erroneous immunoassay findings in the laboratory. Over the years, immunoassay techniques have become crucial in clinical laboratories, where they are widely utilized and serve as the foundation for numerous tests. However, immunoassay interference remains a significant issue in clinical laboratory medicine [1, 2], as it can profoundly impact the precision of analytical results, potentially leading to incorrect diagnoses and inappropriate treatments [1]. This case underscores the critical importance of effective communication between physicians and laboratorians to ensure optimal clinical outcomes. It is essential for each laboratory to establish a personalized interference detection workflow. Accurate measurement of serum estradiol is beneficial for diagnosing infertility, menopause, exogenous estradiol administration, *in vitro* fertilization (IVF) treatment, and estradiol-secreting tumors. For women of reproductive age, falsely elevated estradiol results can lead to unnecessary interventions, significant mental stress, and missed or delayed fertility opportunities. Here, we report the case of a patient who underwent extensive yet ultimately unnecessary medical examinations due to incorrect laboratory results obtained from the Siemens Centaur serum estradiol assay, which were affected by heterophile antibody interference.

## 2 CASE PRESENTATION

A 41-year-old female was referred to the obstetric clinic in July 2023. She reported experiencing abdominal pain and sweating, which persisted for approximately two months. Notably, she exhibited no clinical manifestations, such as hypertension, centripetal obesity, or acne. An abdominal examination did not reveal any palpable masses, and there was no evidence of a palpable liver or autoimmune disease. The patient's past medical history included ovarian cyst exfoliation and breast nodules. At the time of referral, her medications consisted of multivitamins, calcium citrate, and polysaccharide-iron complex capsules. The family history was non-contributory, and thyroid function tests were within normal limits. By August 2023, two months postpartum, the patient was not breastfeeding, and her menstrual cycles had not yet resumed. Due to postpartum abdominal pain, sex hormone testing and an abdominal ultrasound were performed. The abdominal ultrasound results were unremarkable, revealing three follicles in each ovary, with each follicle measuring less than 8 mm in diameter. Sex hormone tests revealed significantly elevated levels of estradiol, while progesterone, luteinizing hormone (LH), follicle-stimulating hormone (FSH), and prolactin remained within normal ranges. Over the subsequent three months, she underwent four tests for estradiol, yielding values ranging from 7534 to 9772 pg/ml. All assessments were conducted using a chemiluminescent immunoassay employing the Siemens Centaur XPT analyzer.

A recent sex hormone test in November 2023 yielded the following results: estradiol, 9103.1 pg/mL (mid-follicular: 19.5-144.2, ovulatory peak: 63.9-356.7, mid-luteal: 55.8-214.2, postmenopausal: 0-32.2); LH, 0.1 IU/L (mid-follicular: 1.9-12.5, ovulatory peak: 8.7-76.3, mid-luteal: 0.5-16.9, postmenopausal 15.9-54.0); FSH, 15.2 IU/L (mid-follicular: 2.5-10.2, ovulatory peak: 3.4-33.4, mid-luteal: 1.5-9.1, postmenopausal: 23-116.3), and prolactin, 6.5 ng/mL (female: 2.8-29.2, postmenopausal: 0.17-4.15). Tumor markers, such as CA125, AFP, CA199, and CEA were also within normal reference intervals. Her serum cortisol levels were normal in both tests, and there were no abnormal findings on the MRI, along with the absence of related clinical symptoms, which effectively ruled out Cushing’s syndrome. The pelvic/transvaginal ultrasound, abdominal computed tomography, and magnetic resonance imaging revealed no adnexal or adrenal masses, and the endometrium was observed to be thin.

Drugs, such as letrozole and norethindrone, were administered to the patient, yet the effect was unsatisfactory. Over the next half a year, the medication-induced amenorrhea in the patient worsened. After a comprehensive review of the patient's clinical condition and a multidisciplinary treatment discussion, the precise diagnosis remained uncertain. This uncertainty raised concerns that laboratory errors may have resulted in falsely elevated estradiol levels. Consequently, the laboratory initiated a primary response program to screen for potential interference. Serum protein electrophoresis and rheumatoid factor (RF) levels were evaluated and found to be normal. The patient's serum sex hormone-binding globulin (SHBG) levels were within the normal range, thus eliminating this as a potential source of interference. While some clinicians recommend a laparoscopic exploration to obtain a definitive diagnosis for elevated estradiol levels and abdominal pain, others advocate for the continuation of medical treatment to manage amenorrhea. Given that the diagnosis had not been confirmed, neither laparoscopic exploration nor medical therapy was deemed appropriate. Therefore, it was essential to reconfirm the estradiol concentrations to eliminate any potential for erroneous results.

## 3 CASE REPRESENTATION

We used four methods to verify whether the falsely elevated estradiol levels were due to the presence of heterophile antibodies. Patient samples were sent to other laboratories (Table **1**) that used a distinct immunoassay method. All analyses were conducted in accordance with the manufacturer’s instructions. The Siemens platform produced a notably distinct result for estradiol compared to other platforms, suggesting that the Siemens Centaur method may be susceptible to analytical interference. The gradient dilution sampling test is a common approach for addressing spurious test results. Consequently, the patient's serum was diluted 5, 10, and 20 times using manufacturer-validated sample diluent to evaluate linearity. Importantly, the 20-fold dilution exhibited no linear correlation with the findings. Polyethylene glycol (PEG) precipitation and blocking studies using a heterophile antibody blocker (HBT) were conducted in the laboratory where the Siemens Centaur method was originally used. The serum was mixed well with an equal volume of PEG fluid, and the mixture was centrifuged at 15,000 × g for 15 minutes. The supernatant was analyzed to determine the concentration of estradiol. Serum samples from two healthy non-pregnant women were collected as controls. Nonetheless, a decrease in estradiol concentration was observed in the patient's sample (Table **2**). The HBT targeted the polyclonal antibody system, which was obtained from Scantibodies Laboratory Inc., and was used to conduct an immunoassay interference analysis. Briefly, 500 μL of the patient sample was pipetted into the bottom of the HBT tube and incubated for 1 hour at room temperature. Subsequently, pre-incubation of the serum sample with an HBT tube resulted in a significant (81.3%) reduction in measured estradiol concentration from 8212 pg/ml pre-HBT to 1532 pg/ml post-HBT (Table **2**).

Based on the experimental results, we concluded the observed interference to be due to heterophile antibodies. Consequently, the aromatase inhibitor letrozole was discontinued. In the following months, the patient exhibited no symptoms or signs, and her menstrual cycle returned to its normal state.

## 4 DISCUSSION

Several interferences in immunoassays have been identified over the years; while some are no longer encountered in routine practice, cross-reaction, heterophile antibodies, biotin, and anti-analyte antibodies continue to pose challenges. It is widely recognized that heterophile antibodies can disrupt immunoassays, particularly in the measurement of hormones, human chorionic gonadotropin (hCG), troponins, and other serological markers [3-7]. Our investigation into this case provided strong evidence for the presence of generalized heterophile antibodies in a 41-year-old female, which interfered with Siemens Centaur immunoassays, resulting in falsely elevated estradiol levels during the postpartum period.

The suspicion of assay interference relies heavily on the expertise of laboratory professionals and clinicians. When interference is suspected, comparing results with alternative immunoassays can be an effective approach. Interference is typically associated with specific classes, subclasses, or isotypes of antibodies, leading to variable outcomes across different techniques. In our case, patient samples were tested for estradiol concentration on four distinct platforms, suggesting that the initially observed high estradiol levels were likely attributable to interference. If feasible, reanalysis using liquid chromatography-tandem mass spectrometry (LC‒MS/MS) should be considered. While immunoassays are currently the most widely used method for measuring clinical analytes, the development of LC‒MS/MS offers a more specialized, accurate, and reliable alternative. However, the technical expertise required for LC‒MS/MS and its limited availability in clinical laboratories contribute to the continued prevalence of immunoassays.

After excluding technical issues and personnel errors, several dilutions of samples should be performed to identify approximately 60% of samples exhibiting interference, characterized by a lack of parallelism and linearity [8]. Notably, in our study, the 20-fold dilution yielded nonlinear results; however, this was not definitive evidence of interference. Consequently, PEG precipitation and blocking studies were conducted. The use of PEG as a reagent has been demonstrated to be effective in producing immunoglobulin-free samples for analytical purposes. It has been demonstrated to be effective in immunoassays for hormones, such as prolactin, thyroxine, triiodothyronine, testosterone, SHBG, estradiol, cortisol, and growth hormone. However, it is crucial to note that PEG cannot be regarded as a universal agent for all immunoassays. In our study, we observed a significant decrease in estradiol concentration following PEG precipitation, and control samples exhibited a non-specific reaction, indicating that the interference may be caused by immunoglobulins. The number of potential errors leading to serious clinical consequences is concerning, given the numerous immunoassays performed. Although rare, the phenomenon of falsely increased estradiol is not negligible. To the best of our knowledge, immune interference caused by heterophile antibodies has been documented in other immunoassays. However, instances of falsely elevated estradiol due to heterophile antibodies are infrequently reported in the literature (Table **3**) [7, 9-15]. While previous studies have explored estradiol interference due to heterophile antibodies, our case adds to the scientific literature by presenting a specific clinical scenario in which such interference notably impacted diagnosis and treatment decisions. This case underscores the importance of considering heterophile antibody interference in patients with atypical hormone levels, particularly in complex clinical situations. As a result, the insights gained from this case will provide valuable guidance for the management of similar abnormal estradiol test results in the future. In recent years, improvements in antibody specificity have led to a decreased focus on cross-reactivity interference caused by endogenous molecules, such as the estradiol structure. Aromatase inhibitors, which lower estrogen levels in the body by inhibiting or inactivating aromatase, are widely utilized in the treatment of estrogen-related diseases, including breast cancer. Drugs with a similar structure to estradiol, specifically fulvestrant [16, 17] and exemestane [18], are most associated with cross-reactivity; however, these were not taken by the patient in the present case.

A study conducted on the Roche test platform has demonstrated norethindrone to exhibit very weak cross-reactivity, while the aromatase inhibitors exemestane, formestane, and letrozole showed no detectable cross-reactivity [19]. This finding is inconsistent with the results of previous studies [16-18], underscoring the need for further investigation to ascertain whether exemestane and formestane interfere with the immunodetection of estradiol. Estrogen is widely recognized for its significant role in the development of the female mammary gland, particularly in the postpartum period [20, 21]. Typically, estrogen levels experience a marked decline following delivery; however, they may remain elevated in certain situations, including psychological factors [22], non-breastfeeding [23], and obesity [24]. Our patient had a previous history of breast nodules and ovarian cyst exfoliation, and she was not breastfeeding. These factors were related to the post-HBT estradiol concentration, which remained above the laboratory reference range. Another possible explanation for the discrepancy may be inadequate blocking.

## CONCLUSION

One of the most significant findings in our study is the critical role of effective communication between physicians and laboratory personnel when laboratory results contradict clinical symptoms. It is essential for each laboratory to establish a personalized workflow for detecting interference. Once immune interference is suspected, this workflow should be activated. Laboratory personnel must pay close attention to such situations and promptly eliminate any interference to ensure the accuracy of the test report.

## Figures and Tables

**Table 1:** Patient estradiol results using different platform on a same serum and methods characteristics of assays.

Laboratory	Platform	Dilution Factor	Estradiol Levels(pg/mL)	Method	Coated Antibody	Labeled Antibody
West China Second University Hospital	Siemens Centaur XP	5	8218	CLIA	Sheep monoclonal antibody	Acridinium ester-labeled
Chengdu First People's Hospital	Beckman DxI800	/	189	CLIA	Sheep monoclonal antibody	Alkaline Phosphatase Conjugated Antibody
West China Hospital, Sichuan University	Roche Cobas E801	/	169	ELCIA	Rabbit monoclonal (Streptavidin)	Ruthenium complex
Sichuan Provincial People's Hospital	Abbott I2000	/	151	CLIA	Rabbit monoclonal antibody	Acridinium ester-labeled

**Note:** /: No data; CLIA: Chemiluminescent Immunoassay; ELCIA: Electrochemiluminescence Immunoassay.

**Table 2:** Estradiol concentration of the patient’s plasma and control samples before (native) and after serial dilution, PEG precipitation, pre-treatment by HBT obtained by Siemens Centaur XPT.

Sample Unit: pg/mL	Patient’s Sample	Control 1	Control 2
Native	8212*	2620	1743
After serial dilution (Dilution factor)	1643.6(5) 795(10) 372.2(20)	/	/
Recovery (%) after PEG precipitation	<22%	93.4%	91.9%
HBT	1532	/	/

**Note:** *: The plasma sample was diluted using the manufacturer’s (Siemens) diluent 5 times.

/: No data; PEG: Polyethylene glycol; HBT: heterophile antibody blocker

**Table 3:** Summary of reported patients whose estradiol levels were interfered.

Author	E_2_ concentration	Detection platform that interferes with estradiol results	Test platform to compare results	Interference Substances	Dilution Testing	HBT Treatment	PEG Treatment	Others
Paul Atkins [7]	8069 pmol/L	Siemens ADVIA Centaur	Abbott^®^ Alinity and Ortho Clinical Diagnostics^®^ Vitros5600 methods	Heterophile antibodies	ND	From 1207 pmol/L pre-HBT to 236 pmol/L post-HBT	ND	Serum protein electrophoresis
Jerome H. Check [9]	1290/544 pg/mL	ES300	Radioimmunoassay	Anti-rabbit antibody	NA	ND	ND	ND
Ellen Anckaert [10]	757pg/mL	Elecsys assays, Roche Diagnostics	Radioimmunoassay	E_2_ was detected after organic solvent extraction	Linear	ND	ND	Organic solvent extraction denatures proteins
Donald L. Gordon [11]	1737 pg/mL	Radioimmunoassay	ELISA	IgA lambda that bound to the 125I-labeled tracer of the E_2_ assay.	ND	NA	ND	Gel electrophoresis
Jing Zhang [12]	619 pg/mL	Siemens ADVIA Centaur	Beckman, DxI 800	Heterophile antibodies	ND	ND	ND	ND
Fabienne Langlois [13]	3073 pmol/L	Roche Cobas 602	liquid chromatography–tandem mass spectrometry;	IgG k gammopathy w	Non-linear	NA	ND	Gel electrophoresis
Gergely Talaber [14]	1106 pmol/L	Siemens ADVIA Centaur	Abbott Alinity and LC-MS/MS	larger hormone-immunoglobulin complex	ND	2184 pmol/L post-HBT	321.8 after PEG	Gel electrophoresis
Kyohei Furusawa [15]	128 pg/mL	Roche cobas e411	LC-MS/MS	Insufficient reagent dose	ND	ND	ND	ND
Our case	8212 pg/mL	Siemens ADVIA Centaur	Beckman DxI800/ Roche Cobas E801/ Abbott I2000	Heterophile antibodies	Non-linear	From 8212pg/mL Pre-HBT to 1532 pg/mL post-HBT	Decreased from 8212 pg/mL to242 pg/mL	ND

**Note:** HBT, heterophilic antibody blocker; PEG, polyethylene glycol; ND, not done; NA, not available.

## Data Availability

The data and supportive information are available within the article.

## LIST OF ABBREVIATIONS

FSH: Follicle-stimulating Hormone;
LH: Luteinizing Hormone;
HBT: Heterophile Antibody Blocker
IVF: *In vitro* Fertilization
RF: Rheumatoid Factor
SHBG: Sex Hormone-Binding Globulin
PEG: Polyethylene Glycol
LC-MS/MS: Liquid Chromatography-tandem Mass Spectrometry
hCG: Human Chorionic Gonadotropin

## STANDARDS OF REPORTING

CARE guidelines were followed.
